# A rare 300 kilometer dispersal by an adult male white‐tailed deer

**DOI:** 10.1002/ece3.7354

**Published:** 2021-03-09

**Authors:** Remington J. Moll, Jon T. McRoberts, Joshua J. Millspaugh, Kevyn H. Wiskirchen, Jason A. Sumners, Jason L. Isabelle, Barbara J. Keller, Robert A. Montgomery

**Affiliations:** ^1^ Department of Natural Resources and the Environment University of New Hampshire Durham NH USA; ^2^ Wildlife Biology Program, W.A. Franke College of Forestry and Conservation University of Montana Missoula MT USA; ^3^ Missouri Department of Conservation Science Branch Columbia MO USA; ^4^ Minnesota Department of Natural Resources St. Paul MN USA; ^5^ Research on the Ecology of Carnivores and their Prey (RECaP) Laboratory Department of Fisheries and Wildlife Michigan State University East Lansing MI USA

**Keywords:** chronic wasting disease, migration, movement ecology, ungulate

## Abstract

Despite the key roles that dispersal plays in individual animal fitness and meta‐population gene flow, it remains one of the least understood behaviors in many species. In large mammalian herbivores, dispersals might span long distances and thereby influence landscape‐level ecological processes, such as infectious disease spread. Here, we describe and analyze an exceptional long‐distance dispersal by an adult white‐tailed deer (*Odocoileus virginianus*) in the central United States. We also conducted a literature survey to compare the dispersal to previous studies. This dispersal was remarkable for its length, duration, and the life history stage of the dispersing individual. Dispersal is typical of juvenile deer seeking to establish postnatal home ranges, but this dispersal was undertaken by an adult male (age = 3.5). This individual dispersed ~300 km over a 22‐day period by moving, on average, 13.6 km/day and achieving a straight‐line distance of ~215 km, which was ~174 km longer than any other distance recorded for an adult male deer in our literature survey. During the dispersal, which occurred during the hunting season, the individual crossed a major river seven times, an interstate highway, a railroad, and eight state highways. Movements during the dispersal were faster (mean = 568.1 m/h) and more directional than those during stationary home range periods before and after the dispersal (mean = 56.9 m/h). Likewise, movements during the dispersal were faster (mean = 847.8 m/h) and more directional at night than during the day (mean = 166.4 m/h), when the individual frequently sheltered in forest cover. This natural history event highlights the unpredictable nature of dispersal and has important implications for landscape‐level processes such as chronic wasting disease transmission in cervids. More broadly, our study underscores how integrating natural history observations with modern technology holds promise for understanding potentially high impact but rarely recorded ecological events.

## Graphical abstract

In November 2017, an adult male white‐tailed deer in seemingly prime condition undertook a remarkable and rare 300 km dispersal over a period of 22 days. We describe and analyze this movement and discuss its implications for dispersal ecology, white‐tailed deer biology, and potential disease transmission.

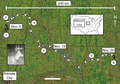

## 1 INTRODUCTION

The spatial scales of animal movement are integral to ecological research and the development of effective conservation and management strategies (Allen & Singh, 2016; Levin, 1992; Lindenmayer et al., 2006; Montgomery et al., 2018; Weins, 1989). Long‐distance movements, such as dispersal and migration, are key fitness‐optimizing behaviors that involve foraging, breeding, and survival trade‐offs (Clobert et al., 2012; Hansson & Åkesson, 2014). These trade‐offs not only influence the fitness of individual organisms but can also shape meta‐population dynamics with subsequent implications for ecosystem functioning (Hanski, 1998; Nathan et al., 2008; Turchin, 1998). From an applied perspective, long‐distance animal movements highlight the importance of landscape‐level approaches to management and conservation because local dynamics can be influenced by events occurring at vast distances (Allen & Singh, 2016; Lindenmayer et al., 2006). Yet, because such events are rare, they are insufficiently documented, difficult to model, and poorly integrated into ecological theory (Clark et al., 2001; Cooper & Marra, 2020; Dixon et al., 2005).

Advancements in animal tracking technologies (Hebblewhite & Haydon, 2010; Millspaugh & Marzluff, 2001) have facilitated remote monitoring of animals over longer periods and at finer spatiotemporal resolutions than ever before (Kays et al., 2015). Consequently, such advancements increase opportunities to document rare movement events that have typically gone undetected due to logistical constraints (Cooper & Marra, 2020; Oyer et al., 2007). When coupled with modern remote‐sensing technology, we can now also describe and analyze such movements with respect to landscape features with unprecedented precision. These detailed descriptions can help elucidate behavioral responses and decisions during long‐distance movements and can inspire novel mechanistic hypotheses regarding their drivers (Clobert et al., 2012; Oyer et al., 2007).

White‐tailed deer (*Odocoileus virginianus*) are large (adult mass: ~40–70 kg), ubiquitous ungulates that inhabit North America and northern regions of South America, acting as a driver of ecosystem functioning and as an important game species throughout their range (Hewitt, 2011; Waller & Alverson, 1997). Typical movement modes of white‐tailed deer include relatively short (i.e., tens to hundreds of meters) daily and weekly movements among stable home ranges, occasional forays outside of these home ranges, dispersal, and, in harsh landscapes, seasonal migration (Clements et al., 2011; DeYoung & Miller, 2011; Oyer et al., 2007). Dispersal is most often undertaken by young males, usually in their second year and is typically driven by social antagonism between dams and their offspring in spring to avoid inbreeding, sexual competition in fall, or some combination thereof (DeYoung & Miller, 2011; Long et al., 2008; Marchinton & Hirth, 1984; Nixon et al., 1994). Long et al. (2008) found that yearling male dispersal in spring was longer than in fall, suggesting that effective inbreeding avoidance requires greater dispersal distances than does a reduction in sexual competition. Less commonly, adult males (i.e., 2.5 years or older) may disperse in fall to maximize the potential of locating a mate (Marchinton & Hirth, 1984), and such movements are hypothesized to be lengthened by local hunting pressure (Sparrowe & Springer, 1970). However, given that adult male dispersal is uncommon and seldom studied, it remains poorly understood (DeYoung & Miller, 2011; Oyer et al., 2007). Often, the dispersal by young individuals is referred to as *natal dispersal* while that of adults is termed *breeding dispersal* (Ronce, 2007).

Because of their high movement rates and frequent contact with conspecifics, adult male white‐tailed deer play an important role in the spread and dynamics of several infectious diseases, including chronic wasting disease (CWD), bovine tuberculosis, and Lyme disease (Bouchard et al., 2013; Clements et al., 2011; Grear et al., 2006; O’Brien et al., 2002). These diseases all carry substantial costs: CWD is a lethal and expanding threat to cervids that is currently known to occur in the United States, Canada, South Korea, and Norway; Lyme disease is the most prevalent vector‐borne zoonotic disease in North America; and bovine tuberculosis is a globally‐distributed disease that can infect humans and a wide range of domestic and wild species (Carlson et al., 2018; Schwartz et al., 2017). Therefore, although long‐distance dispersals of adult male deer are inadequately documented and analyzed, they can play a critical role in landscape‐level processes, especially the spread of infectious diseases.

Here, we report on an exceptionally long‐distance dispersal by an adult male white‐tailed deer in the central United States. Because we did not document the natal range of this individual, we cannot determine whether this was a natal dispersal, a breeding dispersal, or a dispersal‐like movement of another type. We hereafter simply refer to this event as a “dispersal” while acknowledging this uncertainty. We quantified characteristics of this dispersal by coupling GPS collar data collected from the dispersing individual with remote sensing data depicting the landscape through which it moved. To evaluate the uniqueness of this dispersal, we compared it to existing white‐tailed deer dispersals described in the literature. Overall, this dispersal is perhaps the longest ever recorded for an adult male white‐tailed deer. Our study also shows how landscape features generally considered barriers or deterrents to movement (major highways, railroad tracks, and rivers) can ultimately fail to impede long‐distance dispersal. We conclude by discussing the implications of this dispersal in particular and rare events in general for movement ecology and disease transmission.

## 2 MATERIALS AND METHODS

### 2.1 Study area

**FIGURE 1:**
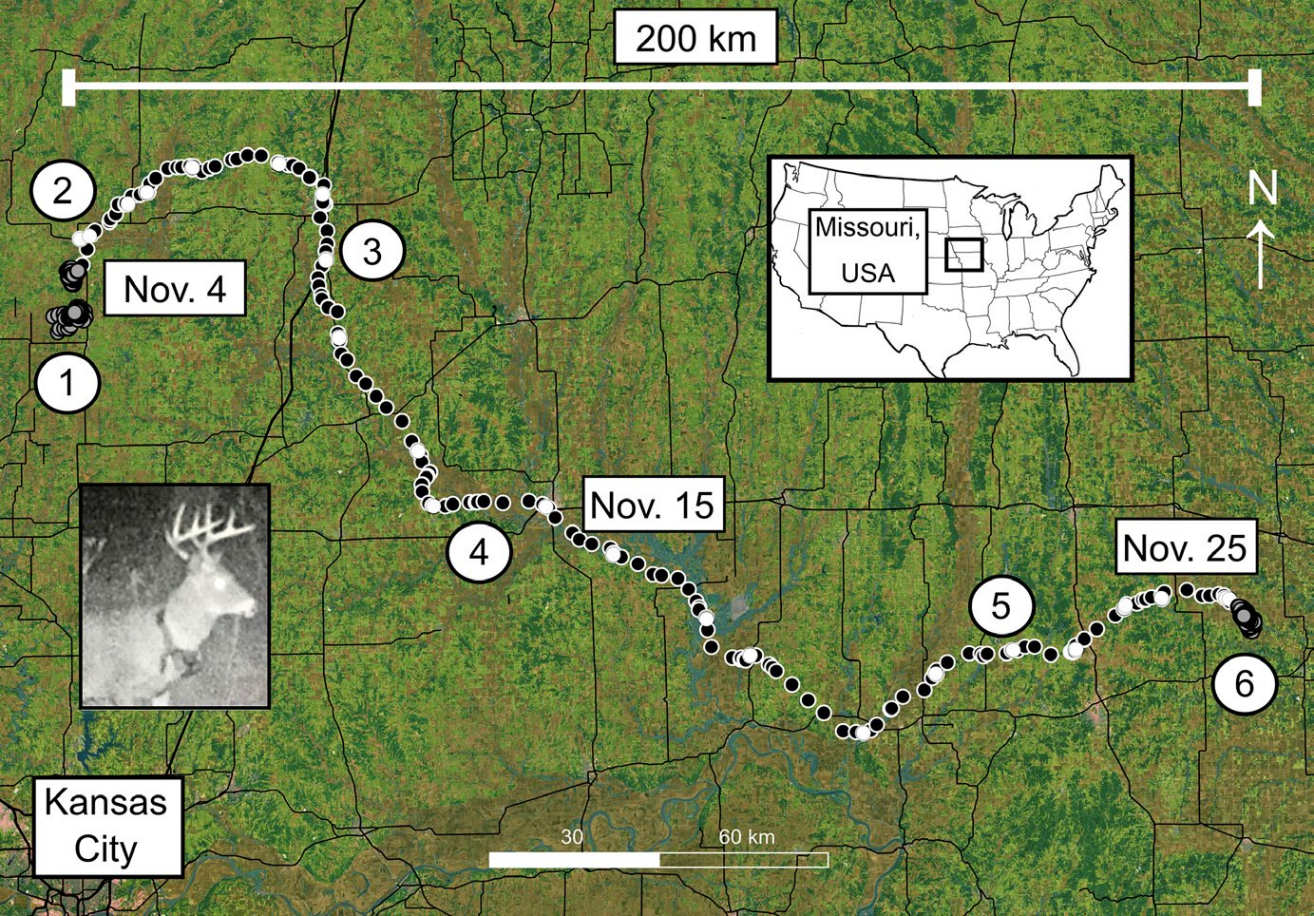
The home ranges and dispersal of N17003, an adult male white‐tailed deer inhabiting northwestern Missouri, USA in 2017–2018. Numbers indicate: (1) initial home ranges (gray circles; see Figure 2a,b), (2) an urban daytime resting/foraging site (see Figure 4a), (3) a major highway crossing (see Figure 4b), (4) a major river crossing (see Figure 4c), (5) a daytime resting/foraging site in a forest patch within an agricultural field (see Figure 4d), and (6) a final home range (gray circles; see Figure 2c). Daytime and nighttime locations are depicted as white and black circles, respectively, and black lines are major roads (primary or secondary highways). N17003 is pictured in the inset, as captured by a camera trap (image: Missouri Department of Conservation)

We positioned our study in a ~6,000 km^2^ area (Figure 1) consisting of a Glaciated Plains ecotype (Wright et al., 2019) characterized by rolling topography (elevation range: 185 to 475 m; United States Department of Agriculture, 2006) and a matrix of agriculture (44.5% of the total area, primarily soybean and corn), grasslands (37.7%), and deciduous forest (12.3%, dominated by oak *Quercus* sp. and hickory *Carya* sp.), with small amounts of interspersed urban areas (2.5%). Most grasslands consist of livestock pasture, with a smaller proportion enrolled in the Conservation Reserve Program (19.1% of grasslands, or 4.6% of the total area), which tends to support native grasses (e.g., big bluestem (*Andropogon gerardi*), little bluestem (*Schizachyrium scoparium*), and switchgrass (*Panicum virgatum*)). The climate in the study area is typical of the mid‐continental United States, consisting of warm and humid summers (mean daily high temperature in July 32.3°C) and relatively cold winters with low to moderate snowfall (mean daily high temperature in January of 4.2°C and mean monthly snowfall of 10 cm; NOAA Online Weather Data; w2.weather.gov/climate/xmacis.php?wfo=eax, accessed August 18 2020).

### 2.2 Field methods

On 26 January 2017, we captured an adult male white‐tailed deer (hereafter referred to as N17003) using a rocket net. This capture was part of a large study in which 343 white‐tailed deer were captured and fitted with GPS collars between January – March of 2015 to 2019. At the time of capture, these included 58 adult males, 50 yearling males, 123 male fawns, 65 adult females, 7 yearling females, and 43 female fawns. All capture and handling procedures were approved by the University of Missouri Institutional Animal Care and Use Committee (approval no. 8216).

During handling, N17003 exhibited no indication of poor health, with no injuries nor any parasites detected. We examined tooth wear and replacement patterns and determined N17003 to be at least 2.5 years old at the time of capture (Severinghaus, 1949). We measured the base neck girth (60 cm), overall body length (186 cm) and recorded the number of points and spread of the antlers (4 and 33.5 cm, respectively). These characteristics are indicative of a young adult male deer in good health (Sauer, 1984).

We equipped N17003 with an Iridium GPS radio‐collar (model G2110E; Advanced Telemetry Systems) that was programmed to attempt a locational fix according to two schedules: (1) every 1.5 hr from October 26 to December 7, and (2) every 5 hr during all other times. This two‐part sampling design was associated with a broader study objective regarding white‐tailed deer movements during the hunting season. Importantly, the period with the higher fix rate coincided with N17003’s dispersal, which facilitated a more detailed analysis of this movement. The collar included a mortality switch that would send a signal to e‐mail and a cell phone after 8 hr of motionlessness. In total, N17003 was under our capture and handling protocols for 10 min. Upon release, N17003 fled while flagging and displayed no signs of distress.

### 2.3 Analysis

We used RStudio version 1.1.463 running R version 3.5.2 (R Core Team, 2017) and QGIS 3.12.1 (QGIS.org, 2020) to analyze the movements of N17003 post‐release. Initial visual inspection of N17003’s locations revealed a clear long‐distance dispersal that occurred during the fall of 2017, with the distinct directional movements beginning on November 4 and concluding on November 25 (Figure 1). This dispersal was preceded and followed by relatively stationary clusters of locations (Figure 1). Accordingly, we calculated home ranges for these periods of stationarity, including (a) the winter, spring, and summer of 2017 (January 27 to September 4), (b) the fall of 2017 (September 5 to November 3), and (c) the fall, winter, spring, and early summer of 2017–2018 (November 26 to June 20). We calculated these home ranges using an autocorrelated Brownian bridge approach to estimate 95% utilization distributions (Bullard, 1991; Horne et al., 2007) via the package adehabitatHR (Calenge, 2015). Individual N17003 died in June 2018 from unknown causes.

To analyze this dispersal, we extracted movement metrics (movement speed and turning angle) from N17003’s locations using the package amt (Signer et al., 2019). We used Tukey post‐hoc tests to compare movement speeds among periods (i.e., dispersal and the three home range periods). Next, we used a *t* test to compare daytime and nighttime movement speeds during the dispersal. We compared turning angles across these periods and within the dispersal using homogeneity of concentration tests (Mardia & Jupp, 2000) via the package circular (Lund & Agostinelli, 2017). These tests evaluate differences in the concentration of turning angles (i.e., the parameter κ of a von Mises distribution), which represents the degree of linearity in movements (Mardia & Jupp, 2000). For all tests, we considered differences to be statistically significant at an *α* = 0.05 level. Finally, to provide a movement‐by‐movement chronological narrative of the dispersal, we analyzed N17003’s locations via visual inspection with respect to landscape features in QGIS using the 2016 National Land Cover Database (Homer et al., 2015) and a Google basemap (Google, 2015).

### 2.4 Literature survey

We used a formal literature survey to compare N17003’s dispersal to those recorded in other studies. On 1 August 2020 we used the Web of Science to search all collections from 1864–2020 using the following Boolean string of terms: **TOPIC:** (white‐tailed deer) *AND* **TOPIC:** (long‐distance OR dispersal OR excursion) *AND* **TOPIC:** (movement). This search yielded 170 studies. We eliminated studies that were either irrelevant (i.e., not a study of white‐tailed deer movement) or did not report movement distances. For each relevant study, we recorded the location and habitat of the study area, number of individuals tracked, method of tracking, maximum reported distance moved by a tracked individual, sex, and age class of that individual, and seasonal timing of that movement. For the maximum reported distance reported in these studies, we recorded the final Euclidean (straight‐line) dispersal distance rather than the length of the movement path, which was not consistently reported and would be subject to considerable variation according to tracking methodology (e.g., lower locational fix rates would reduce movement path length).

## 3 RESULTS

### 3.1 Home range and dispersal characteristics

**FIGURE 2:**
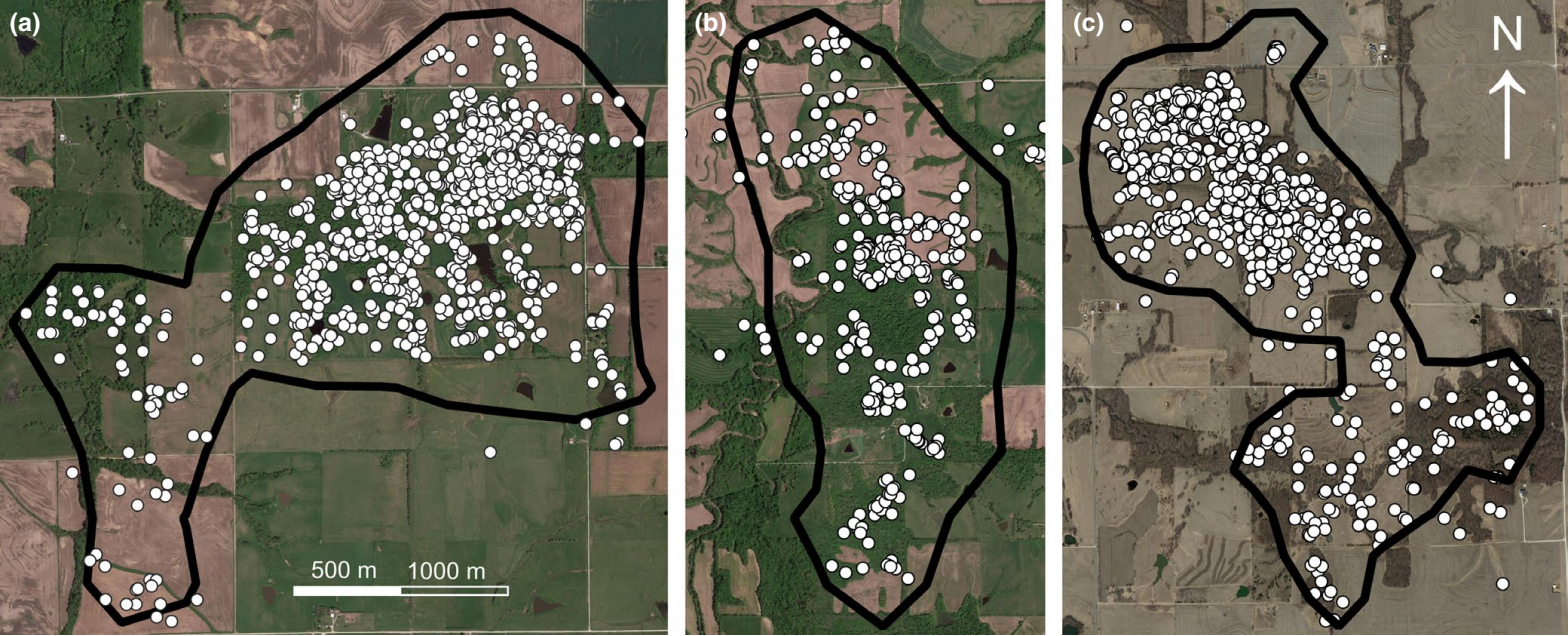
Home ranges of N17003, an adult male white‐tailed deer inhabiting northwestern Missouri, USA in 2017–2018, prior to (a and b) and following dispersal (c). Panel a: the home range from 27 January 2017 to 4 September 2017 was 4.09 km^2^ and consisted of 19.0% forest cover. Panel b: the home range from 5 September 2017 to 3 November 2017 was 3.17 km^2^ and consisted of 39.6% forest cover. Panel c: the home range from 26 November 2017 to 20 June 2018 was 3.21 km^2^ and consisted of 19.3% forest cover

Over 509 days of tracking, we recorded 2,904 locations for N17003. The sizes of N17003’s home ranges prior to and following dispersal were comparable and ranged from 3.17 km^2^ to 4.09 km^2^ (Figure 2). These home ranges also shared similar proportions of forested habitat, which ranged from 0.19 to 0.40 (Figure 2). N17003’s dispersal spanned 22 days from 4 November 2017 to 25 November 2017. During this time, N17003 covered approximately 300 km (i.e., moving ~ 13.6 km/day), resulting in a straight‐line displacement of approximately 215 km (Figure 1).

**FIGURE 3:**
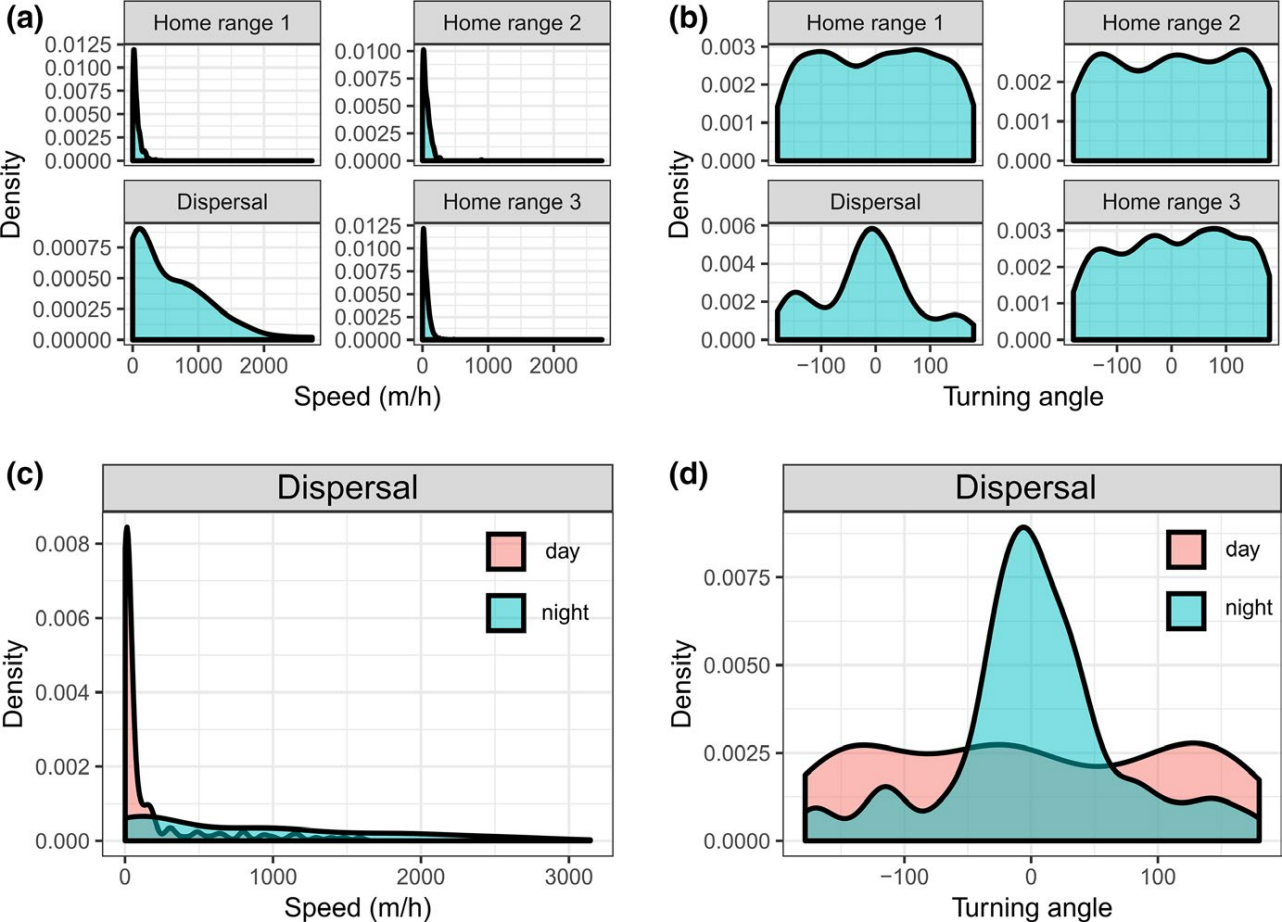
Univariate kernels depicting the movement speeds (panels a and c) and turning angles (panels b and d) of N17003, an adult male white‐tailed deer inhabiting northwestern Missouri, USA in 2017–2018. Home ranges refer to those depicted in Figure 2

During dispersal, N17003’s mean movement speed significantly differed from that during each home range period (all pairwise *p*‐values < .001), but there were no differences in movement speeds among home range periods (pairwise *p*‐values ranged from 0.83 to 0.99; Figure 3a). N17003’s mean movement speed during dispersal was 568.1 m/h (*SD* = 754.0, range = 1.4–3,144.1) compared an average of 56.9 m/h (*SD* = 58.6, range = 0–898.6) across the three home range periods. While dispersing, N17003’s mean movement speed was over five times higher at night (mean = 847.8 m/h, *SD* = 835.7, range = 1.4–3,144.1) than during the day (mean = 166.4 m/h, *SD* = 331.2, range = 1.4–1587.3; *p* <.001; Figure 3c). Turning angles were more concentrated near zero (indicating a tendency for straight‐line movements) during dispersal (κ_disp_ = 0.65) than during any home range period (κ_HR1_ = 0.03, κ_HR2_ = 0.18, κ_HR3_ = 0.09; *p* <.001; Figure 3b). Likewise, while dispersing, N17003’s turning angles were more concentrated near zero at night (κ_night_ = 1.25) than during the day (κ_day_ = 0.23; *p* <.001; Figure 3d). Finally, 56.5% of daytime locations during dispersal fell within a forested cell, compared with 19.7% at night.

### 3.2 Chronological dispersal description

**FIGURE 4:**
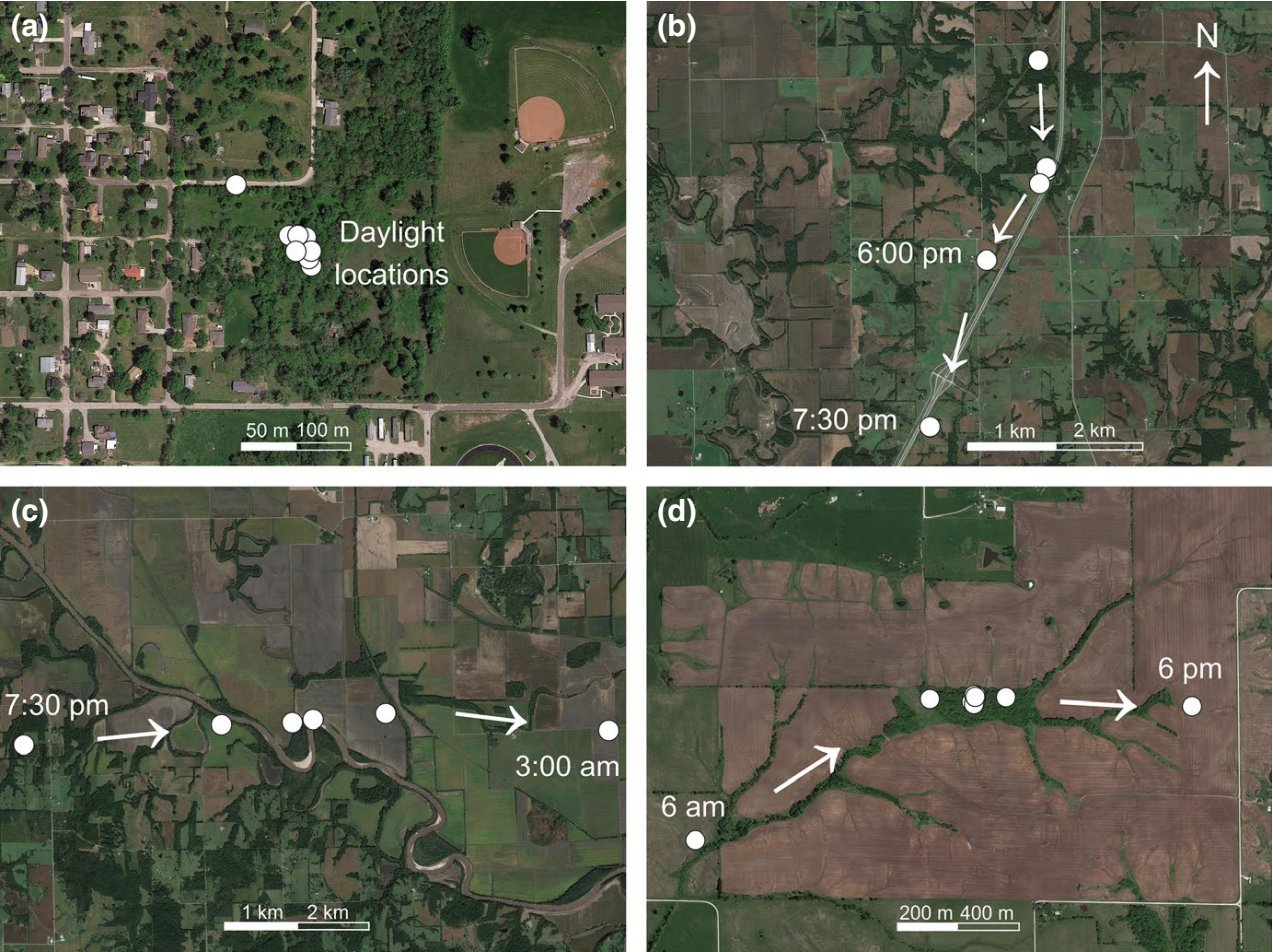
Unique events during the dispersal of N17003, an adult male white‐tailed deer inhabiting northwestern Missouri, USA in 2017–2018. Panels show: (a) an urban daytime resting and foraging location near the beginning of the dispersal; (b) an evening crossing of a major four‐lane highway; (c) a nighttime crossing of a major river; (d) a daytime resting and foraging forest patch surrounded by agricultural fields. Note that scales are unique to each panel and that these events are also depicted in Figure 1

N17003’s dispersal began on 4 November 2017 when he departed his fall 2017 home range (Figure 1, Figure 2b). N17003 moved into the town of Stanberry (population: ~1,000), where he spent the daylight hours in a small suburban forest patch (Figure 4a). N17003 continued northeast, crossing a ~10–20 m wide branch of the Grand River. On November 8, N17003 moved east (Figure 1) and again crossed the Grand River. This eastward trajectory was maintained until November 10, when N17003 encountered the city of Bethany (population: ~3,000) and an adjacent, 4‐lane interstate highway (Figure 1). N17003 then turned south and traveled 14 km before crossing that highway on November 11 between 6:00p.m. and 7:30p.m. (Figure 4b).

N17003 moved south‐southeast and crossed a two‐lane state highway between 3:00a.m. and 4:30a.m. on November 12. N17003 then moved southeast along that highway. On November 13, N17003 crossed the 25–50 m wide Grand River, and then crossed railway tracks the following day between 3:00a.m. and 4:30a.m. N17003 continued east, crossing the Grand River twice more, once between 9:00p.m. and 10:30p.m. on November 14 at a location where its width ranged from 75–100 m (Figure 4c), and again the following morning between 3:00a.m. and 4:30a.m. where its width ranged from 50–75 m. On November 16, N17003 encountered the city of Chillicothe (population: ~10,000) and traversed its southwest corner while crossing an interchange of a four‐lane state highway and a two‐lane state highway between 6:00p.m. and 7:30p.m. N17003 then traveled east, crossed the Grand River for the sixth time between 10:30p.m. and midnight on November 16 at a location where its width was 75–100 m, and followed the river east.

N17003 moved southeast and crossed the Grand River between 3:00a.m. and 4:30a.m. on November 17 at a location where its width was 75–100 m. From November 18 to November 21, N17003 continued east, crossing five two‐lane state highways (Figure 1). During the day on November 21, N17003 remained in a small (<0.1 km^2^) forest patch surrounded by agricultural fields (Figure 4d). Over the last four days of the dispersal, N17003 continued east and crossed two additional highways on consecutive days, one that was four‐lane (between 9:00 p.m. and 10:30 p.m.) and another that was two‐lane (between 6:00 p.m. and 7:30 p.m.). On the evening of November 25, N17003 reached a forest patch where he would establish a final home range, in which he remained until his death on 20 June 2018 (Figure 3c). Evidence at the mortality site suggested N17003 died of hemorrhagic disease, although scavenging and decomposition prevented confirmation as cause of death. Thus, throughout the dispersal, N17003 crossed a major river seven times, an interstate highway, a railroad, and eight state highways.

### 3.3 Literature survey

Our literature survey yielded 15 relevant studies of white‐tailed deer dispersals or long‐distance movements (Table 1). These studies tracked a total of 1761 individual white‐tailed deer (1,084 males, 611 females; sex ratio was not reported 66 individuals), with a mean of 113.6 individuals per study (*SD* = 119.1, range: 1–409). These studies took place across a diversity of landscapes, from those containing very little forest cover (e.g., 1%; Kernohan et al., 1994) to those with contiguous forest (Nelson, 1993). Most studies deployed VHF telemetry, with just three using GPS collars. Across studies that reported the age class distribution of tracked individuals, yearlings were the most often tracked age class (*N* = 865; 85.4% of all individuals) and adult males were the least often tracked (*N* = 25; 2.5%). The longest‐recorded movement from each study averaged 93.1 km (sd: 68.1; range: 6.7–212.6). These movements most frequently occurred in the spring (*N* = 10 studies). The sex of the longest‐moving individual was roughly just as likely to be male (53.3% of studies) as female (46.6%). These individuals were much more frequently yearlings (76.9% of studies) than adults (23.1%), reflecting the overall emphasis on tracking yearlings. The longest recorded movement was a 212.6 km spring dispersal by a yearling male in an agricultural South Dakota landscape with 1% forest cover (Kernohan et al., 1994). The longest recorded movement for an adult male was 41 km (Nixon et al., 1994). In two studies, the movement path of a dispersing individual was considerably longer than the maximum straight‐line dispersal distance. Oyer et al. (2007) reported on a yearling female that moved a total of 462 km over 1.5 years with a final straight‐line dispersal distance of approximately 41 km. Similarly, Lutz et al. (2016) reported on a yearling female that moved 258 km over 55 days with a final straight‐line dispersal distance of approximately 35 km.

**TABLE 1:** Results from a literature survey conducted in August 2020 to document long‐distance movements by white‐tailed deer (*Odocoileus virginianus*). All study locations were within the USA. For studies that reported percentage of forested habitat, nonforest habitat was either agriculture, grassland, or developed land. Distance is the final maximum straight‐line dispersal or long‐distance movement recorded and Season is the season in which that dispersal or long‐distance movement occurred. Abbreviations: *N* = number of individuals tracked; GPS = global positioning system; RT = radiotelemetry; TRR = tag‐release‐return; Y = yearling; A = adult

Study location	Habitat	*N*	Method	Distance (km)	Season	Sex	Stage	Ref
Nebraska & Iowa	40% forest	50	RT	6.7	Spring	F	NA	1
Texas	Shrub rangeland	23	RT	7.3	Winter	M	Y	2
Pennsylvania	49%–57% forest	9	GPS	10.2	Spring	M	Y	3
Minnesota	forest	63	TRR	35.2	NA	M	A	4
Wisconsin	80% forest	409	RT	35.8	Spring	M	Y	5
Wisconsin	Predominately forest	1	RT	41	Spring	F	Y	8
Pennsylvania	60%–88% forest	229	RT & GPS	52.9	Spring	F	Y	6
Maryland	50% forest	51	RT	56	NA	M	Y	7
Minnesota	Mixed prairie – forest	57	TRR	88	NA	F	A	4
Illinois	2%–3% forest	264	TRR	100–120	Spring	NA	Y	9
Wyoming	foothills	66	GPS	106.6	Spring	F	NA	10
Nebraska & Iowa	40% forest	85	RT	121	Spring	M	Y	11
Illinois	2%–20% forest	267	TRR	161	NA	M	Y	12
Minnesota	Contiguous forest	79	RT	168	Spring	F	Y	13
Minnesota	Agriculture (3% forest)	77	RT	205	Spring	F	A	14
South Dakota	1% forest	31	TRR	212.6	Spring	M	Y	15

Note

References: 1 (Hygnstrom et al., 2011), 2 (McCoy et al., 2005), 3 (Long et al., 2010), 4 (Carlsen & Farmes, 1957), 5 (Peterson et al., 2017), 6 (Lutz et al., 2016), 7 (Rosenberry et al., 1999), 8 (Oyer et al., 2007), 9 (Nixon et al., 1991), 10 (Edmunds et al., 2018), 11 (Clements et al., 2011), 12 (Nixon et al., 1994), 13 (Nelson, 1993), 14 (Brinkman et al., 2005), 15 (Kernohan et al., 1994).

## 4 DISCUSSION

We detected one of the longest dispersals ever recorded for white‐tailed deer. Dispersal in white‐tailed deer occurs across sexes and age classes but is most common in young males that seek to reduce inbreeding avoidance, sexual competition, or a combination thereof (DeYoung & Miller, 2011; Long et al., 2008; Marchinton & Hirth, 1984; Sparrowe & Springer, 1970). Such dispersals are typically on the order of tens of kilometers (Table 1; see Long et al., 2005 for an additional meta‐analysis of male white‐tailed deer dispersal distances), although longer movements (i.e., hundreds of kilometers) have been recorded in landscapes with little to no forest cover (Kernohan et al., 1994; Long et al., 2005). In contrast to these trends, N17003 dispersed ~300 km over the course of 22 days for a total straight‐line displacement of approximately 215 km in a landscape with modest (i.e., 10%–15%) forest cover. Moreover, N17003’s dispersal was also remarkable in that it crossed numerous barriers (i.e., rivers, highways, and a railroad) and occurred during an archery and firearms hunting season in a state with more than 500,000 white‐tailed deer hunting permit‐holders (Keller et al., 2017). Therefore, while the observational nature of this study limits our ability to definitively evaluate the proximal drivers of N17003’s movement, it nonetheless remains clear that this dispersal was an exceptionally rare natural history event with important implications for white‐tailed deer ecology and management and the influence of rare events in ecology more generally.

Here, we explore several potential contributing factors that might have influenced N17003’s dispersal while acknowledging the observational limitations of inference. Given that N17003 appeared to be a healthy, 3.5+‐year‐old male at the time of this dispersal, we suggest it was unlikely that social antagonism from genetically related individuals was a proximate driver for movement. In the absence of this social antagonism, two alternative drivers might have influenced the dispersal: sexual competition and hunting pressure. During N17003’s pre‐dispersal movements, he moved several kilometers north in September 2017 for a relatively brief (~two month) period in a new temporary home range before dispersing on November 4 (Figure 1; Figure 2a,b). Conception rates in the study area peak in early to mid‐November (K. Wiskirchen pers. comm.); thus, the dispersal initiation might have been stimulated by sexual competition. Additionally, archery hunting season began in the study area on September 15 and firearms season began on November 11. Therefore, hunter activity in this period also could have stimulated N17003 to shift home ranges and eventually disperse, while the modest level of forest cover in the landscape might have further encouraged these movements. Altered movement patterns due to hunting pressure have often been observed in white‐tailed deer (Kilpatrick & Lima, 1999; Root et al., 1988; Sparrowe & Springer, 1970). The possibility that hunting pressure influenced N17003’s movements is further supported by the observation that throughout the dispersal he tended to remain mostly stationary in forest cover during the day and performed extensive, higher‐speed, and more linear movements at night when hunting pressure was absent (Figure 1; Figure 3). Reduced daytime movements to decrease detectability and elevated nighttime movements are common responses of white‐tailed deer to hunting pressure (Kilgo et al., 1998; Little et al., 2016; Sullivan et al., 2018). CWD can also influence white‐tailed deer movement (Edmunds et al., 2018), but we have no indication that this individual had the disease, nor had CWD been detected in the immediate area surrounding the movement path.

N17003’s dispersal highlights the multi‐scale nature of animal movement. The spatial scales of animal movement and habitat selection have been long‐recognized to be hierarchical (Johnson, 1980) and adaptive movements across such scales enable individuals to navigate heterogeneous landscapes (McPeek & Holt, 1992; Nathan et al., 2008). We documented distinct scales of movement related not only from home range to dispersal to home range, but also within the dispersal itself (Figure 1; Figure 3). Dispersal movement was faster and more linear compared with home range movement in much the same manner as nighttime movement during dispersal was faster and more linear than in the daytime (Figure 3). These patterns imply that the scales at which landscape heterogeneity interact with animal movements change not only across seasons, but also across days and weeks. N17003’s path also illustrates the importance of short‐term refugia, or resting places, along a given dispersal route. For example, several very small forest patches (i.e., <0.1 km^2^) appeared to play important roles as stop‐over habitat, sometimes in the midst of surrounding matrices that lacked cover as a result of agricultural (Figure 4d) or urban (Figure 3a) development. In addition to these localized habitat patches, linear features, including major highways and rivers, appeared to influence directional trajectory of movements at broad spatial scales during the dispersal (Figure 1). Depending on the landscape context, cities, roads, and rivers can all act as movement barriers for white‐tailed deer (Peterson et al., 2017; Robinson et al., 2012). In the case of N17003, the study area's major river presented little impediment to movement, while roads and cities appeared to influence, but not obstruct, the overall dispersal trajectory (Figure 1).

Long‐distance movements can play a crucial role in landscape‐level ecological processes including infectious disease transmission (Altizer et al., 2011; Russell et al., 2005). For example, long‐distance movements involving dispersal or migration can directly lead to the initial establishment and subsequent spread of disease (Altizer et al., 2011; Clobert et al., 2012). In our study system, a disease of major management and public concern is chronic wasting disease (CWD), a fatal neurological disease in cervids. CWD is rare but spreading in the state of Missouri (Keller et al., 2017), a scenario reflective of many other states (Carlson et al., 2018). Given that CWD is spread via direct contact and environmental contamination (Carlson et al., 2018), and that adult males are a key vector (Clements et al., 2011), long‐distance dispersals such as the one recorded here represent a potential management challenge. Such movements enlarge the spatial scales of disease monitoring while heightening the importance of coordination across agencies and legislative entities (Edmunds et al., 2018). Additionally, while we consider N17003’s dispersal to be rare, we also acknowledge that much remains unknown about the prevalence of such movements. Tracking studies typically only document the movements of a tiny subset of populations; in a state such as Missouri with >1 million deer (J. Isabelle, pers. comm.), long‐distance dispersals might be rarely documented in practice but fairly commonplace in reality across larger populations. Moreover, extensive movements (hundreds of kilometers) in dispersing yearling females have been documented in at least two cases despite relatively modest final dispersal distances (i.e., ~30–40 km; Oyer et al., 2007; Lutz et al., 2016). Further, as noted above, adult males are more rarely tracked than other age classes, often due to logistical challenges associated with collaring and animal harvest (Millspaugh et al., 2012). Another possibility is that adult dispersals are observed but not reported in the literature. Given that CWD management plans are informed by the spatial scale of animal movements (Carlson et al., 2018), obtaining accurate information on the frequency and nature of movements like those reported here is important.

More broadly, our study highlights the importance of documenting rare ecological events and using the lessons learned from such instances to update knowledge and generate new hypotheses. Observations of rare, long‐distance movements have also been made for other large mammal species (e.g., moose *Alces alces* and cougars *Puma concolor*; Hoffman et al., 2006; Thompson & Jenks, 2005). In these and other cases, individuals dispersing abnormally long distances tend to be juvenile males seeking to avoid inbreeding or reduce competition. However, exceptions to these trends exist. For example, Bowles and Gladfelter (1980) recorded a 3‐year‐old male moose that dispersed ~900 km and Stoner et al. (2008) reported on a female cougar that moved over 1,300 km to achieve a straight‐line dispersal of 357 km. In both cases, the causes of such rare movements were uncertain and hypothesized to be the result of numerous, interactive factors related to population density, resource availability, hunting pressure, and landscape fragmentation. Technological advancements in animal‐borne loggers and satellite‐derived imagery has transformed our ability to document such events with high precision across spatio‐temporal scales unimaginable just a few decades ago, thereby holding promise for disentangling the multiple contributing mechanisms (Kays et al., 2015; Moll et al., 2007). Translating such observations into improvements in ecological theory and applied practice requires care but represents an important avenue of future work. As in other disciplines that often employ advanced statistical models (e.g., economics), the prevalence and impact of rare events, and of fat‐tailed distributions of general processes, has often been overlooked in empirical study (Clark et al., 2001; Dixon et al., 2005; Taleb, 2007). At the same time, recent trends in ecological publishing have tended to deemphasize careful documentation of rare or previously unknown natural history events (Moore et al., 2020). New initiatives focused on natural history by several major ecological journals have responded to this challenge by encouraging detailed explorations of such events (Moore et al., 2020). Moving forward, the integration of natural history with advanced technology and statistical modeling holds promise for understanding the uniqueness of ecological events and their contribution to the predictive and explanatory generality of our discipline.

## 5 ETHICS STATEMENT

All animal capture and handling procedures were approved by the University of Missouri Institutional Animal Care and Use Committee (approval no. 8216).

## CONFLICT OF INTEREST

None declared.

## AUTHOR CONTRIBUTION

**Remington J Moll:** Conceptualization (lead); Formal analysis (lead); Writing‐original draft (lead); Writing‐review & editing (lead). **Jon McRoberts:** Conceptualization (equal); Data curation (equal); Investigation (equal); Project administration (equal); Writing‐review & editing (equal). **Joshua Millspaugh:** Conceptualization (equal); Funding acquisition (lead); Investigation (equal); Methodology (equal); Project administration (lead); Supervision (lead); Writing‐review & editing (equal). **Kevyn Wiskirchen:** Investigation (equal); Methodology (equal); Project administration (equal); Writing‐review & editing (equal). **Jason Sumners:** Project administration (equal); Writing‐review & editing (equal). **Jason Isabelle:** Investigation (equal); Methodology (equal); Project administration (equal); Writing‐review & editing (equal). **Barbara Keller:** Investigation (equal); Methodology (equal); Project administration (equal); Supervision (equal); Writing‐review & editing (equal). **Robert Montgomery:** Conceptualization (supporting); Funding acquisition (supporting); Investigation (equal); Project administration (supporting); Supervision (supporting); Writing‐review & editing (equal).

## Data Availability

The data associated with this article are deposited on the Dryad Digital Repository (https://doi.org/10.5061/dryad.qfttdz0g5).
